# Management of a Symptomatic Maxillary Implant Adjacent to the Schneiderian Membrane Using Cone Beam Computed Tomography (CBCT)-Guided Explantation and Reimplantation: A Case Report

**DOI:** 10.7759/cureus.113962

**Published:** 2026-08-04

**Authors:** Keily J Portillo Avila, Richard M Cavero, Ana M Moreno Marin, Yaniet E Lorenzo Rivero, Marilyn Gomez Rodriguez

**Affiliations:** 1 Periodontics, Dental Loft, San Pedro Sula, HND; 2 General Practice, Vedha Family Dentistry, New Jersey, USA; 3 Dentistry, Signature Dental Group Delray Beach, Florida, USA; 4 Dentistry, Natural Smiles, Florida, USA; 5 Dentistry, Dr. Bob Pediatric Dentist, Miami, USA

**Keywords:** cbct, collagen membrane, dental implant, explantation, implant complications, implant reimplantation, maxillary sinus, schneiderian membrane, symptomatic implant, three-dimensional planning

## Abstract

Dental implants are a predictable treatment option for the replacement of missing teeth; however, complications related to implant positioning and proximity to adjacent anatomical structures may result in discomfort and compromise long-term outcomes. Cone beam computed tomography (CBCT) plays an important role in the diagnosis and management of implant-related complications by providing detailed three-dimensional evaluation of implant position and surrounding anatomy.

This case report describes the management of a symptomatic maxillary implant in a patient who presented with intermittent pain during mastication and occasional discomfort in the region of a previously restored implant-supported crown. The patient subsequently reported accidental ingestion of the implant crown and was referred to a hospital to rule out aspiration or airway involvement. Following medical evaluation, CBCT imaging was obtained and revealed a wide-diameter implant positioned in close proximity to the Schneiderian membrane.

Based on the clinical and radiographic findings, treatment consisted of CBCT-guided explantation of the existing implant followed by site redevelopment using bone grafting and a collagen membrane. Three-dimensional digital planning was performed to optimize the position, angulation, and dimensions of the replacement implant while maintaining a safe relationship with the Schneiderian membrane. A narrower implant was subsequently placed according to the digital treatment plan.

Postoperative healing was uneventful. After six months of osseointegration, a second-stage procedure was performed, and a healing abutment was placed for soft tissue maturation. A digital impression was obtained using a scan body, and a definitive implant-supported crown was fabricated and delivered. At follow-up, the patient reported complete resolution of symptoms, satisfactory function, and improved comfort during mastication. Clinical and radiographic evaluation demonstrated successful implant integration and stable peri-implant tissues. This case highlights the value of CBCT-guided diagnosis, explantation, and reimplantation in the management of symptomatic implants located near the Schneiderian membrane and demonstrates the importance of three-dimensional planning in achieving predictable functional and restorative outcomes.

## Introduction

Dental implants are considered a predictable and effective treatment option for the replacement of missing teeth, demonstrating high long-term survival rates and favorable functional and esthetic outcomes. Successful implant therapy depends on accurate diagnosis, careful treatment planning, and proper positioning in relation to adjacent anatomical structures. In the posterior maxilla, limited bone availability and the proximity of the maxillary sinus present unique challenges that may increase the risk of complications when implant placement is not optimally planned [1-6].

The Schneiderian membrane lines the maxillary sinus and plays an important role in sinus health and regeneration. Implants positioned in close proximity to this structure may present diagnostic and treatment planning challenges, particularly when associated with unfavorable implant dimensions or angulation. However, proximity alone does not establish a causal relationship with patient symptoms, making comprehensive clinical and radiographic evaluation essential. Clinical evaluation alone may be insufficient to accurately assess the relationship between an implant and surrounding anatomical structures, making three-dimensional imaging an essential component of diagnosis and treatment planning [7-11].

Cone beam computed tomography (CBCT) has become an indispensable tool in implant dentistry because it provides detailed visualization of bone anatomy, implant position, and proximity to critical structures. CBCT allows clinicians to identify implant-related complications, evaluate available bone volume, and develop more precise surgical and restorative treatment plans. Digital implant planning further enhances treatment predictability by facilitating the selection of appropriate implant dimensions and ideal positioning while minimizing the risk of anatomical complications [12-16].

This report describes the management of a symptomatic maxillary implant located in close proximity to the Schneiderian membrane. Treatment involved CBCT-guided diagnosis, explantation of the existing implant, site redevelopment with bone grafting and guided bone regeneration, and subsequent placement of a new implant using three-dimensional digital planning. The clinical outcome demonstrates the value of advanced imaging and digital treatment planning in the management of implant-related complications in anatomically challenging areas [17-20].

## Case presentation

A 25-year-old female presented with intermittent pain during mastication and occasional discomfort associated with a previously restored implant-supported crown in the left posterior maxilla. The patient reported that the symptoms had gradually increased over time and negatively affected her comfort during function. Her medical history was noncontributory, and no systemic conditions contraindicating implant therapy were identified.

**Figure 1:**
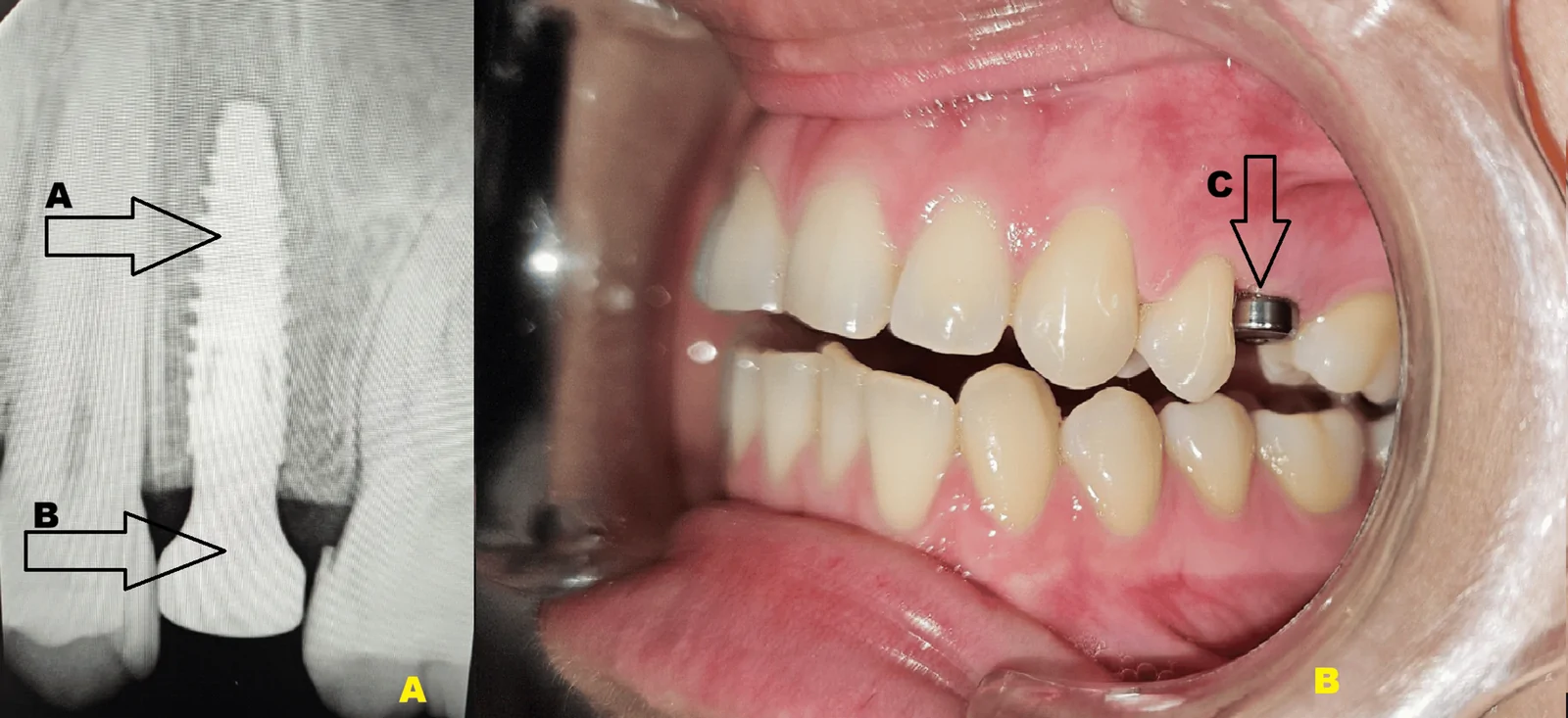
Initial radiographic and clinical presentation of the symptomatic maxillary implant (A) Periapical radiograph showing the implant fixture in the left posterior maxilla. (B) Implant abutment connected to the implant after loss of the implant-supported crown. (C) Clinical view of the implant site demonstrating the exposed abutment following crown loss.

Clinical and radiographic examination revealed a maxillary implant restoration in the affected region. The patient reported accidental ingestion of the implant-supported crown and was referred for medical evaluation to rule out aspiration or airway involvement. Following confirmation that no respiratory complications were present, further dental evaluation was performed. Clinical examination demonstrated the exposed implant abutment after loss of the crown, while radiographic assessment confirmed the presence of the implant fixture in the posterior maxilla (Figure 1).

**Figure 2:**
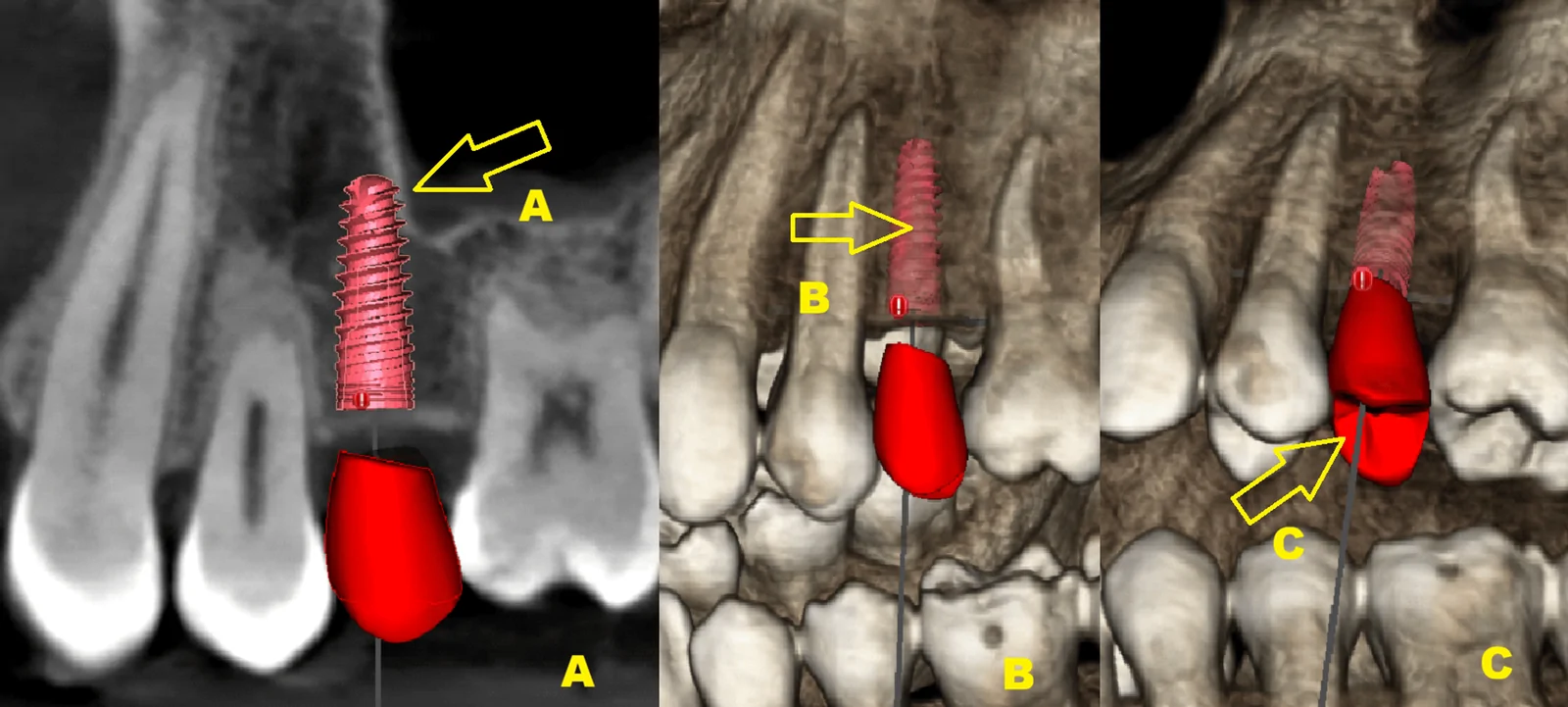
CBCT evaluation and digital planning for implant replacement (A) Cross-sectional CBCT view demonstrating virtual placement of a standard-diameter implant to replace the previously removed wide-diameter implant while maintaining a safer anatomical position. (B) Three-dimensional reconstruction showing the planned implant position in relation to the Schneiderian membrane. (C) Prosthetically driven digital planning illustrating the ideal position and angulation of the future implant-supported crown.

Given the patient's persistent symptoms, cone beam computed tomography (CBCT) was obtained to evaluate the implant and surrounding anatomical structures. Three-dimensional imaging revealed a wide-diameter implant positioned in close proximity to the Schneiderian membrane. The original CBCT was not calibrated to obtain a precise measurement of the distance between the implant apex and the Schneiderian membrane. Based on the available imaging, the implant appeared adjacent to the sinus floor; however, the relationship could not be quantified objectively. Based on the clinical and radiographic findings, removal of the existing implant was recommended. Digital treatment planning was subsequently performed to determine the ideal position, angulation, and dimensions of the replacement implant while maintaining a safe anatomical relationship with the Schneiderian membrane. A standard-diameter implant was selected to replace the previously placed wide-diameter implant (Figure 2).

**Figure 3:**
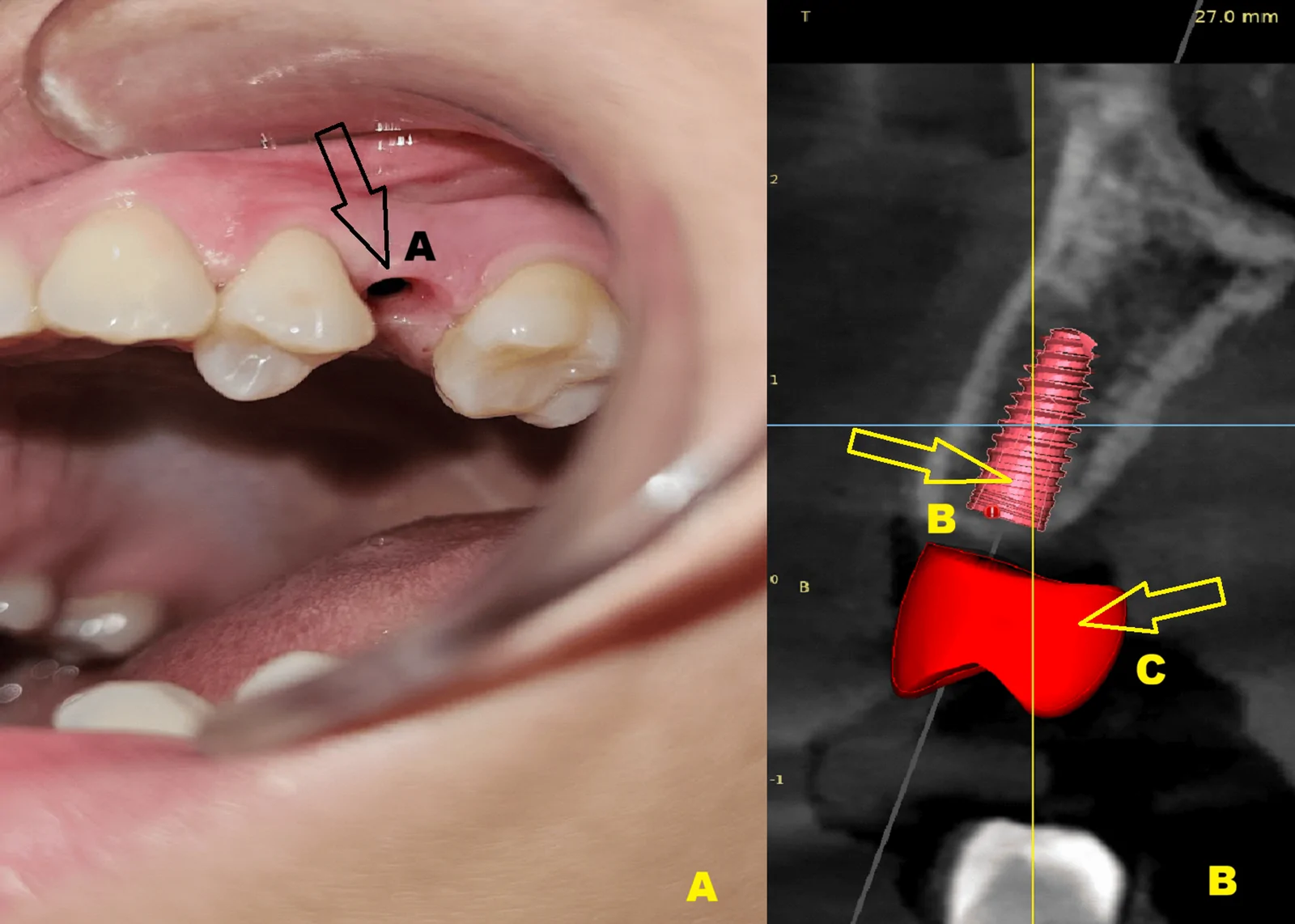
Implant placement and prosthetic planning following site redevelopment (A) Clinical view showing the newly placed implant in the left posterior maxilla after healing and implant insertion. (B) Cross-sectional CBCT image demonstrating the position and angulation of the newly placed implant within the regenerated site. (C) Digital prosthetic planning illustrating the design and position of the future implant-supported crown.

The existing implant was atraumatically removed, and the site was allowed to heal appropriately before reimplantation. Following healing, the replacement implant was placed according to the digital treatment plan. Clinical examination demonstrated satisfactory implant positioning and soft tissue healing. Postoperative CBCT evaluation confirmed appropriate implant angulation and placement within the available bone while maintaining a favorable relationship with the surrounding anatomical structures. Virtual restorative planning was also performed to optimize the position and emergence profile of the future implant-supported crown (Figure 3). 

**Figure 4:**
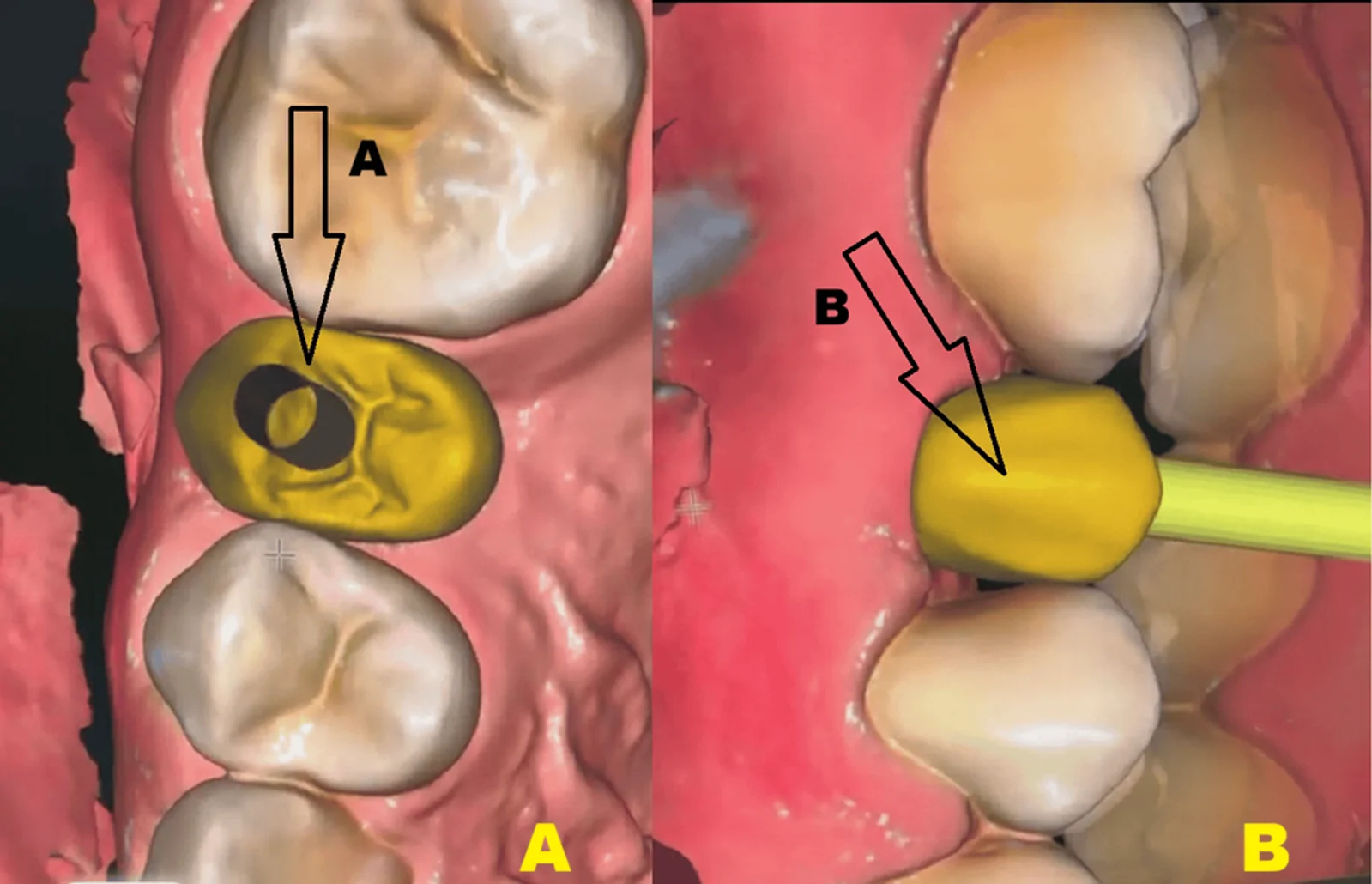
Digital prosthetic planning using a scan body and virtual crown design (A) Digital intraoral scan with a scan body connected to the implant, allowing three-dimensional visualization of implant position, occlusal relationships, proximal contacts, and surrounding anatomical structures for prosthetic planning. (B) Virtual design of the definitive implant-supported crown based on the digital workflow. The restoration was planned to achieve proper occlusal function, proximal contacts, emergence profile, and harmonious integration with the adjacent dentition while respecting the surrounding anatomical structures.

After successful osseointegration, a digital impression was obtained using a scan body connected to the implant. The digital workflow enabled precise visualization of implant position, occlusal relationships, proximal contacts, and surrounding anatomical structures. A definitive implant-supported crown was designed using computer-aided design software to optimize function, esthetics, proximal contacts, and occlusal harmony while respecting the adjacent dentition and anatomical structures (Figure 4). 

**Figure 5:**
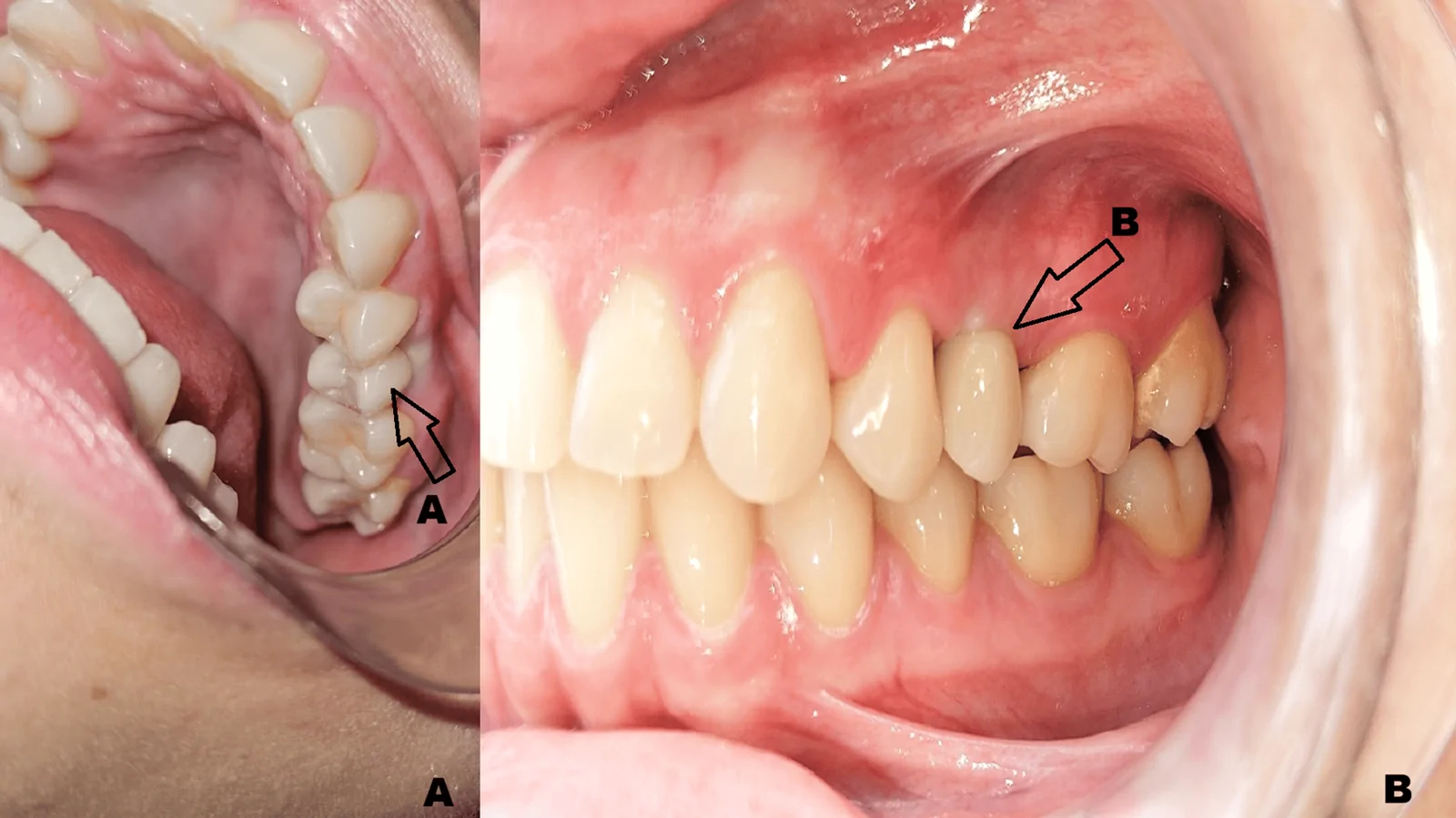
Final clinical outcome after implant-supported rehabilitation (A) Occlusal view of the definitive implant-supported crown demonstrating satisfactory integration with the adjacent dentition, appropriate occlusal anatomy, and harmonious restorative contours. (B) Buccal view of the definitive implant-supported crown showing favorable esthetic integration, soft tissue adaptation, and color matching with the adjacent natural teeth.

The definitive implant-supported crown was subsequently fabricated and delivered. Clinical evaluation demonstrated satisfactory adaptation of the restoration, healthy peri-implant soft tissues, and harmonious integration with the adjacent dentition. Occlusal and buccal views confirmed favorable restorative contours, appropriate occlusion, and satisfactory color matching. At follow-up, the patient reported complete resolution of symptoms and expressed a high level of satisfaction with the functional and esthetic outcome of treatment (Figure 5).

## Discussion

Dental implant therapy is recognized as a highly predictable treatment modality for the replacement of missing teeth, with reported long-term survival rates exceeding 90% in many clinical situations [1,2]. Despite these favorable outcomes, complications related to implant positioning, prosthetic design, and proximity to anatomical structures may compromise patient comfort and treatment success. In the posterior maxilla, limited bone availability and the close relationship between implants and the maxillary sinus represent important considerations during treatment planning.

The Schneiderian membrane plays a critical role in maintaining maxillary sinus health. Although implants positioned in close proximity to the sinus floor or Schneiderian membrane have been associated with discomfort and other complications in some reports, proximity alone does not establish a causal relationship. In the present case, CBCT demonstrated that the implant was located adjacent to the Schneiderian membrane; however, the original imaging did not permit an objective measurement of the implant-to-membrane distance. Therefore, the patient's symptoms can only be described as being associated with the implant position rather than being definitively caused by it [3,4]. In the present case, the patient reported intermittent pain during mastication despite the absence of obvious clinical signs of infection. CBCT evaluation revealed a wide- diameter implant positioned in close proximity to the Schneiderian membrane, providing valuable information that could not be fully appreciated through conventional two-dimensional imaging alone.

CBCT has become an indispensable diagnostic tool in implant dentistry because it allows accurate three- dimensional visualization of implant position, available bone volume, and surrounding anatomical structures [5]. Previous studies have demonstrated that CBCT improves diagnostic accuracy and facilitates safer treatment planning in anatomically challenging regions such as the posterior maxilla [6]. In the present case, CBCT imaging was essential for identifying the unfavorable implant position and guiding the decision-making process.

When implant complications occur, treatment options may include monitoring, prosthetic modification, surgical correction, or implant removal and replacement. The decision depends on the severity of symptoms, implant stability, prosthetic considerations, and the relationship of the implant to adjacent anatomical structures [7-10]. Because the existing implant was associated with persistent symptoms and demonstrated an unfavorable position relative to the Schneiderian membrane, explantation followed by site redevelopment and replacement was considered the most predictable treatment approach.

Digital implant planning has significantly improved the accuracy and predictability of implant placement. Virtual planning allows clinicians to evaluate implant dimensions, angulation, restorative requirements, and anatomical limitations before surgery is performed [11,12]. In the present case, digital planning facilitated the selection of a narrower implant and an improved three-dimensional position, allowing maintenance of a safer distance from the Schneiderian membrane while supporting an ideal restorative outcome.

The use of a digital restorative workflow further contributed to treatment success. Digital impressions obtained with a scan body allowed precise visualization of implant position and facilitated fabrication of a definitive restoration with favorable occlusal relationships, proximal contacts, and esthetic integration. Previous studies have reported that digital workflows can improve efficiency, accuracy, and patient satisfaction in implant-supported rehabilitations [13-20].

Following explantation, guided bone regeneration, reimplantation, and digital restorative rehabilitation, the patient experienced complete resolution of symptoms and reported a high level of satisfaction with the final outcome. Clinical and radiographic evaluations demonstrated successful osseointegration, stable peri-implant tissues, and restoration of function. This favorable result highlights the importance of comprehensive diagnosis and treatment planning when managing implant-related complications.

This case emphasizes the value of CBCT-guided diagnosis, digital implant planning, and prosthetically driven treatment concepts in the management of a symptomatic implant located adjacent to the Schneiderian membrane. Although the patient's symptoms resolved following explantation and replacement, this single case demonstrates only an association between implant location and the reported symptoms and does not establish a direct causal relationship. Careful evaluation of the implant's relationship to adjacent anatomical structures and appropriate implant selection may help optimize treatment planning, reduce complications, and improve long-term clinical outcomes.

## Conclusions

This case illustrates the value of CBCT-guided diagnosis combined with three-dimensional digital planning in the management of a symptomatic implant located adjacent to the Schneiderian membrane. Comprehensive radiographic assessment facilitated safe explantation, site redevelopment, and placement of a replacement implant in a more favorable position, supporting a predictable restorative outcome.

The patient experienced complete resolution of symptoms, successful osseointegration, stable peri-implant tissues, and high satisfaction with treatment. Although symptom improvement was observed following treatment, this report demonstrates an association rather than a proven causal relationship between implant location adjacent to the Schneiderian membrane and the presenting symptoms. This case highlights the importance of comprehensive three-dimensional evaluation and prosthetically driven treatment planning to optimize functional and restorative outcomes in anatomically challenging areas of the posterior maxilla.
